# Implementation of HIV non-occupational post-exposure prophylaxis for men who have sex with men in 2 cities of Southwestern China

**DOI:** 10.1097/MD.0000000000027563

**Published:** 2021-10-29

**Authors:** Yufei Wu, Qiuying Zhu, Yuejiao Zhou, Shujia Liang, Rongjian Li, Nengxiu Liang, Chunying Li, Guanghua Lan

**Affiliations:** Division of HIV/AIDS Control and Prevention, Guangxi Zhuang Autonomous Region Center for Disease Control and Prevention, Nanning, Guangxi, China.

**Keywords:** community-based organization, HIV, men who have sex with men, non-occupational post-exposure prophylaxis

## Abstract

Non-occupational post-exposure prophylaxis (nPEP) has often relied on the joint work of emergency physicians and infectious disease specialists in busy emergency departments and human immunodeficiency virus (HIV)/sexually transmitted infections clinics abroad, where adherence education and follow-up are invariably reactive. In our pilot study, community-based organizations (CBOs) were invited to together implement the nPEP tailored to men who have sex with men (MSM) in 2 cities of Guangxi in Southwestern China, of which experiences and lessons drawn from would be provided to the promotion of nPEP in China.

The study population enrolled MSM individuals prescribed nPEP from September 2017 to December 2019. One-to-one follow-ups by CBOs were applied through the treatment. Predictors of treatment completion were assessed by logistic regression.

Of 271 individuals presented for nPEP, 266 MSM with documented treatment completion or non-completion, 93.6% completed the 28-day course of medication. Completion was associated with reporting side effects (aOR = .10; 95% CI: 0.02–0.38; *P* = .001). The follow-up rate of 91.9% was achieved based on the definition of loss to follow-up. No documented nPEP failures were found, although 1 MSM subsequently seroconverted to HIV due to ongoing high-risk behavior.

CBOs’ engagement in HIV nPEP, especially the “one-to-one” follow-up supports by peer educators partly ensure adherence and retention to nPEP. Tailored interventions are needed to address the subsequent high-risk behaviors among the MSM population.

## 1 Introduction

Over 30 years, China's human immunodeficiency virus (HIV) epidemic has kept increasing that contributed to 0.958 million survival cases as of October 2019,^[1]^ but disparities remain across various regions. Most HIV diagnoses occurred in southwest China, which comprises largely cases from Sichuan, Yunnan, and Guangxi.^[2]^ The fast escalating epidemic among men who have sex with men (MSM) is of particular concern in recent years.^[3]^ Overall, HIV prevalence among the MSM group was approximately 8% in 2015, with a three-time higher prevalence observed in five provinces including Guangxi of southwest China, according to a scoping review.^[4]^ In 2017, among newly diagnosed cases in China, 25.5% were MSM,^[5]^ and this figure rapidly rose from the rate of 2.5%, reported in 2006.^[6]^ In addition, studies in China show that 2% to 5% of sexually active men have sex with other men in the nation, accounting a total of 2 to 8million male population across the country.^[7]^

Neighboring on the provinces of Guangdong in the east, Hunan in the northeast, and Yunnan in the west, Guizhou in the northwest, the Guangxi Zhuang Autonomous Region (Guangxi) links to Hong Kong and Macao by the waterway of the Xijiang River and shares a borderline of 637 kilometers in the southwest with Vietnam. Due to its location along a major heroin trafficking route connecting Guangxi with Yunnan and Vietnam as well as its close proximity to the world's major heroin-producing base, famous for the Golden Triangle, HIV transmission in Guangxi was triggered initially by intravenous drug use.^[8]^ In 1996, the first case of HIV infection was discovered among local intravenous drug users in Pingxiang city of Guangxi, bordering Vietnam.^[9]^ After then, HIV prevalence via injecting drugs climbed and accounted for 69% of the total reported cases across the region in 2003.^[10]^ With passing years, sexual transmission becomes the dominant mode of HIV spread in Guangxi.^[11]^ Guangxi has occupied the second position in the largest number of reported HIV cases in China.^[12,13]^ MSM are now recognized as a disproportionately affected group in this area of which the proportion of HIV infections among MSM increased from 0.1% in 2005 to 7.27% in 2018 in a total reported number.^[14]^ HIV surveillance data published by Guangxi Center for Disease Control and Prevention (CDC) indicated a trend of rapid HIV incidence among MSM in recent years that HIV positive rate among them ascended from 3.9% in 2010 to 9.67% in 2018.^[15]^

HIV non-occupational post-exposure prophylaxis (nPEP) is a 28-day prescription of antiretroviral therapy (ART) provided within 72 hours of exposure to prevent the infection.^[16–18]^ nPEP is an evidence-based HIV prevention strategy that has been implemented in most countries for decades, of which lessons drawn from suggesting its completion rates are of major concern.^[19]^ Meta-analyses indicate 25.7% to 67.2% of patients who accepted nPEP completed the full course.^[20,21]^ nPEP awareness, access (eg, cost and where available), side effects (SEs), follow-up retention, and drug regimens are associated with lower rates of nPEP completion.^[22–25]^ As a result, the efficacy of nPEP may depend on a comprehensive health service mechanism, including sustainable antiretroviral adherence education, SEs observation, psychological, and follow-up supports during the implementation. In many countries, nPEP services have often relied on the joint work of emergency physicians and infectious disease specialists (IDS) in busy emergency departments and HIV/sexually transmitted infections (STI) clinics, where compliance education and follow-up are invariably reactive.^[26,27]^ A prospective study suggested that funded additional staff could support counseling and follow-up activities.^[28]^

Over the years, nPEP in China has been limited to some surveys on awareness and demands^[29,30]^ and to date, there has not been a comprehensive guideline developed due to lack of domestic research data, despite clinicians in some areas tried to offer nPEP.^[31]^ Supported by the “Technical Collaboration with the People's Republic of China on Innovative Approach on AIDS between U.S. CDC” and “Guangxi Natural Science Foundation,” a cooperative pilot program of HIV nPEP tailored to MSM in 2 cities of Guangxi in Southwestern China was established in 2017, by taking experience and lessons abroad. We invited gay-oriented community-based organizations (CBOs) as our partner to involve in this culturally appropriate and lesbian, gay, bisexual, and transgender (LGBT)-friendly program, given CBOs helpful to retaining MSM in care and treatment in the public health system and decreasing stigma^[32]^ while non-community-based care providers difficultly reach the hidden population.^[33]^ The program addressed nPEP through linkage to counseling, treatment, and follow-up management by close cooperation between CBOs, CDCs/hospitals, and pharmacies. Specifically, we described the efficacy through medication adherence, follow-up retention, and HIV seroconversion among MSM consulting for nPEP under this model. Experiences and lessons drawn from the analysis would be provided to the promotion of nPEP in China.

## 2 Methods

We conducted research from September 2017 to December 2019 in Nanning and Liuzhou cities in Guangxi, southwest China, based on the aforementioned survey^[29]^ finding that MSM in the 2 cities had potential demands for HIV nPEP. Our study was carried out with reference to international nPEP guidelines, including WHO, Canadian, and American guidelines.^[34–37]^

### 2.1 Inclusion and exclusion criteria

MSM, who were 18 years or older, tested HIV-negative, lived in Nanning and Liuzhou. Eligibility assessment should be based on the HIV status of the source whenever possible and may include consideration of background prevalence and local epidemiological patterns. Exposures that may warrant nPEP include: (1) having sex without condoms (including homosexual and heterosexual behaviors), (2) sharing needles with HIV-infected drug users, (3) blood, semen, genital secretions, blood-stained saliva, and wound exudate splashing to eye, nose, and oral cavity or damaged mucosa. Exposures that do not require nPEP include: (1) when the exposed individual is HIV already positive, (2) when the source is established to be HIV negative, and window period is excluded, (3) exposures to bodily fluids that do not pose a significant risk, that is, tears, non-blood-stained saliva, urine, and sweat, and (4) exposure time exceeds 72 hours.

### 2.2 Recommended regimens

HIV nPEP should be offered and initiated as early as possible in all individuals with an exposure that has the potential for HIV transmission, and ideally within 72 hours. A full 28-day prescription of antiretrovirals (ARVs) would be provided for nPEP following the initial risk assessment.^[38]^ Participants should bear the cost of medication sold by commercial pharmacies or pharmacies within hospitals, and doctors in the research explained all regimens available and specifications to them before the prescription. Participants would choose the preferred and affordable regimen:

Regimen 1: Tenofovir fumarate/emtricitabine (TDF/FTC) 300/200 mg once daily + raltegravir (RAL) 400 mg twice daily,^[39]^ RMB 3960 ($ 573.91) for a full 28-day prescription.
Regimen 2: Tenofovir fumarate/emtricitabine (TDF/FTC) 300/200 mg once daily + dolutegravir (DTG) 50 mg once daily,^[36]^ RMB 3960 ($ 573.91) for a full 28-day prescription.
Regimen 3: Elvitegravir/cobicistat/emtricitabine/tenofovir alafenamide (E/C/F/TAF) single tablet once daily,^[19]^ RMB 2980 ($ 431.88) for a full 28-day prescription.
Regimen 4: Tenofovir fumarate/emtricitabine (TDF/FTC) 300/200 mg once daily,^[34]^ RMB 1980 ($ 286.96) for a full 28-day prescription.
According to the availability of medications locally, alternatives would be made with reference to relevant guidelines, for example, abacavir/lamivudine/dolutegravir (ABC/3TC/DTG) 50/600/300 mg combination tablet once daily,^[40]^ RMB 2880 ($ 417.39) for a full 28-day prescription.

### 2.3 Laboratory testing and follow-up

Baseline testing: (1) patients being initiated on nPEP should be tested for HIV antibodies and antigens, fourth-generation screening and (2) tests for syphilis serology, hepatitis B screen, and hepatitis C antibody, complete blood count (CBC), renal, and liver function. Follow-up testing: (1) repeating HIV serology at week-4 to week-6 and 3 months after exposure as well as at 6 months after exposure if hepatitis C infection was acquired from the exposure and (2) repeating CBC, renal, and liver function at week-4 to week-6 after exposure. Other testing: those tested positive for HIV were referred to CDC for western-blot, CD4 count, and viral load tests, and started on ART.

### 2.4 Determination of completing a full 28-day medication, loss to follow-up

Adherence to nPEP: Information on treatment completion was self-reported by the patients at subsequent follow-up or via phone call by CBO staff for conforming. We defined patients who completed 28 days of treatment as adherent. Loss to follow-up (LTFU): Patients had scheduled follow-up at 4 to 6 weeks, 3 months post-exposure as well as 6 months if hepatitis C infection was acquired from the exposure. LTFU was determined if patients did not return to CBOs for HIV tests at least once within the follow-up schedule.^[26]^

### 2.5 Measures to guarantee adherence and retention in nPEP

(1) Training on adherence to medication for CBOs was provided by ART clinics of CDC and hospitals time after time, which covered risk assessment of HIV exposure, medication use, and ways to improve adherence and retention in care. (2) Budget was specially developed for supporting CBOs to carry out compliance education. Participants who showed lab testing reports during follow-up visits obtained RMB 100 ($ 14.49) for transportation. (3) CBOs used the standardized form for documenting nPEP regimens, adherence, SEs, and follow-up tests. A reminder system was established between peer educators and patients, reminding participants to take drugs and return for HIV tests on time though phone calls and social media, for example, WeChat, QQ. (4) The informed consent form included the provisions of drug adherence and follow-up that participants should abide.

### 2.6 Study procedures

To scale up nPEP awareness, we made community publicity and advocacy before the recruitments by the platform of MSM CBOs – Nanning Yitongxing Healthcare Center and Liuzhou Hongying Working Group, and Nanning/Liuzhou CDCs in the 2 cities as well as the provincial CDC of Guangxi. Online publicity via social media of WeChat, MicroBlog, Blued, QQ, and offline advocacy through cards, posters, and brochures were implemented to address nPEP knowledge, and inform the way of seeking services.

**Figure 1:**
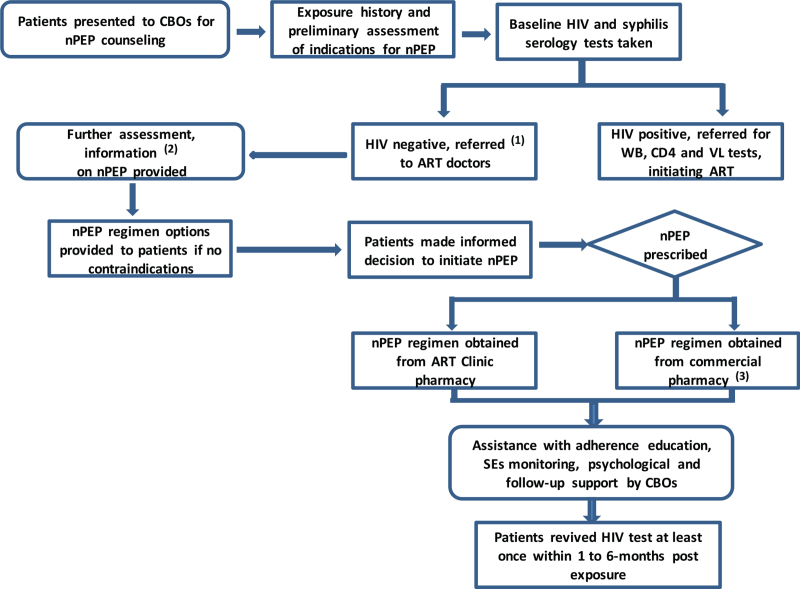
Study flow (1) intra-city transportation from CBOs to ART clinics takes about 15 minutes. (2) Information on the risk of acquiring HIV, safe sex behaviors, risks, and benefits of nPEP were provided. (3) Patients had the option of purchasing prescribed regimens in commercial pharmacy if were in short supply in ART clinic pharmacy. ART = antiretroviral therapy, CBO = community-based organization, HIV = human immunodeficiency virus, nPEP = non-occupational post-exposure prophylaxis.

A collaboration mechanism between CBOs, CDC/hospitals, and pharmacies were established. Firstly, ART clinic doctors of CDC and hospitals organized training on research protocol for MSM peer educators, emphasizing adherence and psychological support, monitoring of SEs, retention in care, and laboratory tests. As the first providers contacted participants, patients’ consulting for nPEP in CBOs was available 24/7 on the protocol basis, which trained peer educators conducted a preliminary risk assessment for MSM to be consulted after exposure, including HIV rapid test of a 4th-generation antibody/antigen combo assay.^[35]^ For those who met eligible recruits, CBOs referred them to ART clinics of CDC and/or hospitals for further risk assessment and baseline testing. For patients seeking nPEP out of normal working hours, CBOs would refer them to on-call physicians in the ART clinic of hospitals. With prescriptions given by doctors, pharmacies in hospitals, or commercial pharmacies would sell medications to patients. One-to-one service was encouraged in our study, that is, the first counselor (peer educator) who received the patient would then accompany him for referral service to ART clinics, and subsequently, assist doctors in offering follow-up services included adherence education, SEs monitoring, psychological support, and follow-up testing throughout the nPEP. In addition, CBOs were responsible for the registration of medical records which covered patients’ basic information, the outcome of the risk assessment, drug uptake, and results of laboratory tests (Fig. 1).

### 2.7 Data collection and analysis

Data were extracted from clinical records onto standardized casebooks, structured according to the Chinese national guidelines for diagnosis and treatment of HIV/AIDS.^[40]^ Information was extracted from the records of the initial consultation and follow-up visits and included: sociodemographic characteristics, risk profile including exposure type that led to nPEP, time of exposure to nPEP, types of ARV prescribed, and the occurrence of SEs, treatment completion, and laboratory test results. Data were stored in the EPI 3.1 Database (EpiData Association, Odense, Denmark).

Outside China, the findings of the previous systematic review have highlighted that adherence remains a challenge across populations including MSM, female sex workers, and victims of sexual assault.^[21]^ However, associated factors impacting adherence have yet to be fully explored,^[41]^ and adverse events of treatment are a well-known reason for nPEP withdrawal in studies on mixed groups.^[21,25,42–44]^ For this study we analyzed the demographics, correlates of SEs associated with nPEP among the only MSM group, and predictors of adherence extended to sociodemographic characteristics, knowing HIV status of source contact, Ses, substance abuse, and nPEP frequency, to further verify whether completion rate for nPEP and associated factors, when prescribed between CBOs, CDCs/hospitals, and pharmacies, are in line with or go beyond previous reports.

For our first aim to assess factors associated with treatment completion vs. non-completion, we performed bivariate analyses using χ^2^ test and Fisher exact test and to evaluate the difference between the 2 groups on socio-demographic, knowing HIV status of the source patient, SEs, substance abuse, and nPEP frequency. Wilcoxon–Mann–Whitney rank-sum test was conducted for continuous variables or abnormal distributions of data, as appropriate. Significant variables were then analyzed in multivariate logistic regression (odds ratios, 95% confidence intervals) to evaluate independent factors associated with adherence to nPEP. Variables with *P* value <.30 in the bivariate analyses were further considered for inclusion in the model. All data analyses were completed using IBM SPSS Statistics 23 (IBM Corporation, Armonk, NY, USA). We considered a two-tailed alpha error of 0.05 throughout the analysis.

## 3 Results

From September 2017 to December 2019, a total of 370 MSM presented LGBT-friendly CBOs for nPEP counseling in the two cities, 281 of them eligible for nPEP uptake, and 271 were enrolled in the study while 9 abandoned the treatment on account of drug burden, 1 rejected to join in the research; 89 were not qualified for nPEP, including 15 presented later than 72 hours after exposure, 4 baseline HIV positive and referred to CDC, 46 evaluated as no risk of HIV acquisition (5 source contacts’ HIV negative, out of window period), 22 only received counseling but refused HIV tests, and 2 were 17 years old, not meet the age for the study.

### 3.1 Socio-demographics and exposure characteristics

A total of 271 participants ranged from 18 to 56 years old (M = 29.8, SD = 7.3), and 21 to 40 years old occupied dominantly of 84.1% (n = 228). 86.3% (n = 234) self-reported as single. More than half of the participants were Han ethnic group (68.6%, n = 186), and 90.4% (n = 261) were from Guangxi. Most patients sought nPEP for the reason of unprotected anal intercourse (UAI) between men and 3.7% (n = 10) experienced condom failure, defined as a broken, torn, or slipped-off condom. 17.0% (n = 46) were with a known HIV-positive source (Table 1).

**Table 1:** Socio-demographic and exposure characteristics of MSM uptake nPEP in Southwestern China (N = 271).

Characteristics	n	%
Age group (years)		
≤20	19	7.0
21–	228	84.1
>40	24	8.9
Marital status		
Single	234	86.3
Married	33	12.2
Divorced	4	1.5
Ethnic groups		
Han	186	68.6
Ethnic minorities	85	31.4
Residency status		
Guangxi	245	90.4
Out of Guangxi	22	8.1
Unknown	4	1.5
Source contact's HIV status		
Positive	46	17.0
Negative (window period not excluded)	8	3.0
Unknown	217	80.1
Exposure type		
Insertive anal (“1”)	19	7.0
Receptive anal (“0”)	89	32.8
Insertive/receptive anal (“0.5”)	3	1.1
Unsure anal position	149	55.0
Insertive/receptive oral	11	4.1
Condon use		
Not use	261	96.3
Torn/slipped	10	3.7

### 3.2 nPEP medication uptake

A 89.7% (243/271) MSM consulted nPEP at CBOs during the daytime while 7.4% (20/271) at 0:00 to 6:00 and 26.9% (73/271) during 18:00 to 0:00. All patients were prescribed nPEP within 72 hours. The time between exposure and medication prescription was less than 24 hours in 177 (65.3%) episodes, 24 to 48 hours in 76 (28.0%) episodes, and 48 to 72 hours in 18 (6.6%) episodes. A 6.3% (17/271) initiated ART at 0:00 to 6:00, and 38.4% (104/271) during 18:00 to 0:00. A 50.9% (138/271) participants were prescribed with TDF/FTC, 34.3% (93/271) and 7.7% (21/271), respectively, with ABC/3TC/DTG and E/C/F/TAF. A 88.6% (240/271) the first time presented for nPEP, and 11.4% (31/271) were nPEP repeat presenters (Table 2). A total of 18 were with documented SEs reporting, with nausea, headache, fatigue, and lethargy/malaise being the most commonly reported. In bivariate analysis, we found no statistically significant association between age, marital status, residency status, ethnic groups or substance/stimulants abuse, time to initiate ART, frequency of nPEP uptake, types of ARV prescribed, and SEs to nPEP; however, treatment completion (*P* = .006) was found statistically significant associated with SEs by the χ^2^ test (Table 3).

**Table 2:** Intervention, follow-up, and compliance to nPEP among MSM in Southwestern China (N = 271).

Characteristic	n	%
Time to receive counselling in CBOs		
0:00–	20	7.4
6:00–	60	22.1
12:00–	110	40.6
18:00–	73	26.9
Unknown	8	3.0
Time to initiate ART		
0:00–	17	6.3
6:00–	30	11.1
12:00–	112	41.3
18:00–	104	38.4
Unknown	8	3.0
Exposure to nPEP time		
<24 h	177	65.3
24–	76	28.0
48–72	18	6.6
Types of ARV prescribed		
TDF + FTC	138	50.9
ABC + 3TC + DTG	93	34.3
E/C/F/TAF	21	7.7
TDF + FTC + DTG	15	5.5
TDF + FTC + RAL	4	1.5
nPEP repeat presenters		
Twice	27	10.0
The third time	2	0.7
The fourth time	2	0.7
None	240	88.6
Side effects		
Yes	18	6.6
No	244	90.0
Unknown	9	3.3
Baseline HIV tests results		
Negative	265	97.8
Positive	0	0.0
Results not sure	6	2.2
HIV test results at week-4–week-6 after exposure		
Negative	237	87.5
Positive	0	0.0
Tests not done	34	12.5
HIV test results 3–6 months after exposure		
Negative	216	79.7
Positive	1	0.4
Tests not done	54	19.9
Follow up (at least once within 1 to 6 months post-exposure)		
Yes	249	91.9
No	22	8.1

**Table 3:** Correlates of SEs associated with nPEP among MSM in Southwestern China (N = 262).

Factors	SEs n (%)	Non-SEs n (%)	*P*	Overall (N = 262)
Age group (years)				
≤20	0 (0.0)	18 (100.0)	.768	18
21–	17 (7.7)	203 (92.3)		220
>40	1 (4.2)	23 (95.8)		24
Marital status				
Single	14 (6.2)	212 (93.8)	.366	226
Married	4 (11.8)	30 (88.2)		34
Divorced	0 (0.0)	2 (100.0)		2
Residency status				
Guangxi	15 (6.4)	221 (93.6)	.222	236
Out of Guangxi	2 (9.1)	20 (90.9)		22
Unknown	1 (25.0)	3 (75.0)		4
Ethnic groups				
Han	9 (4.9)	173 (95.1)	.070	182
Ethnic minorities	9 (11.3)	71 (88.7)		80
Knowing source contact was HIV positive				
Yes	3 (7.0)	40 (93.0)	1.000	43
Unknown	15 (6.8)	204 (93.2)		219
Substance or stimulants abuse				
Yes	2 (3.6)	54 (96.4)	.378	56
No	16 (7.8)	190 (92.2)		206
Time to initiate ART				
0:00–	2 (8.7)	21 (91.3)	.311	23
6:00–	4 (14.3)	24 (85.7)		28
12:00–	7 (6.4)	102 (93.6)		109
18:00–	5 (4.9)	97 (95.1)		102
Frequency of nPEP uptake				
The first time	17 (7.6)	206 (92.4)	.824	223
Twice	1 (3.1)	31 (96.9)		32
Third time	0 (0.0)	5 (100.0)		5
Fourth time	0 (0.0)	2 (100.0)		2
Types of ARV prescribed				
TDF/FTC	9 (6.9)	122 (93.1)	.314	131
ABC/3TC/DTG	5 (5.4)	87 (94.6)		92
E/C/F/TAF	1 (5.0)	19 (95.0)		20
TDF/FTC + DTG	2 (13.3)	13 (86.7)		15
TDF/FTC + RAL	1 (25.0)	3 (75.0)		4
Treatment completion				
Yes	14 (5.6)	234 (94.4)	.006	248
No	4 (33.3)	8 (66.7)		12^∗^

### 3.3 Adherence to nPEP and factors independently associated with adherence

Five MSM could not be confirmed whether or not completed the full course of ART due to loss of contact. 266 (98.2%) MSM with documented treatment completion or non-completion, and 93.6% (249/266) of them completed the 28-day course of medication. For the patients discontinued treatment, 7 due to sex partners being tested negative and 5 without specific reasons. Three stopped treatments because of medication SEs. One patient defaulted treatment on account of busy work and 1 could not afford a whole ART course. In a multivariate model, including age, residency status, and SEs (of which *P* < .3 in univariate analysis), only SEs retained a statistically significant association with adherence to nPEP (aOR = .10; 95% CI: .02–.38; *P* = .001) (Tables 4 and 5).

**Table 4:** Predictors of adherence to nPEP among MSM in Southwestern China.

Factors	Treatment completion n (%) 249 (93.6)	Treatment non-completion n (%) 17 (6.4)	*P* ^∗^	Overall (N = 266)
Age group (years)				
≤20	16 (84.2)	3 (15.8)	.191	19
21–	210 (94.2)	13 (5.8)		223
>40	23 (95.8)	1 (4.2)		24
Marital status				
Single	214 (93.4)	15 (6.6)	1.000	229
Married	33 (94.3)	2 (5.7)		35
Divorced	2 (100.0)	0 (0.0)		2
Residency status				
Guangxi	225 (93.7)	15 (6.3)	.266	240
Out of Guangxi	21 (95.5)	1 (4.5)		22
Unknown	3 (75.0)	1 (25.0)		4
Ethnic groups				
Han	173 (94.5)	10 (5.5)	.419	183
Ethnic minorities	76 (91.6)	7 (8.4)		83
Knowing source contact was HIV positive				
Yes	43 (93.5)	3 (6.5)	1.000	46
Unknown	206 (93.6)	14 (6.4)		220
SEs				
Yes	14 (77.8)	4 (22.2)	.020	18
No/unknown	235 (94.8)	13 (5.2)		248
Substance or stimulants abuse				
Yes	54 (94.7)	3 (5.3)	1.000	57
No	195 (93.3)	14 (6.7)		209
nPEP uptake the first time				
Yes	212 (93.4)	15 (6.6)	1.000	227
No	37 (94.9)	2 (5.1)		39

**Table 5:** Factors associated with adherence to nPEP (N = 266).

Predictors	Crude OR (95% CI)	*P*	Adjusted OR (95% CI)	*P*
Age group (years)				
≤20	1			
21–	0.20 (0.04–1.08)	.061	0.21 (0.04–1.12)	.068
>40	0.19 (0.02–2.40)	.200	0.21 (0.02–2.62)	.228
Residency status				
Guangxi	1			
Out of Guangxi	0.91 (0.10–7.98)	.931		
Unknown	5.96 (0.41–85.95)	.190		
SEs				
Yes	1			
No/unknown	0.10 (0.03–0.42)	.002	0.10 (0.02–0.38)	.001

### 3.4 Laboratory tests, seroconversion, and follow-up retention

At the baseline testing, all participants received HIV tests before nPEP uptake, 97.8% (265/271) were found negative, and 6 (2.2%) MSM showed uncertain HIV antibody test results but were confirmed as negative while further testing was applied (Table 2). A 95.9% (260/271) received syphilis TP rapid tests and 10 MSM were found positive, of which 6 were new infections and referred to STI clinics for treatment. A 82.3% (223/271) had tests for CBC, renal, and liver function. 70.1% (190/271) received hepatitis B tests, 12 were found positive for HBsAg but no liver abnormalities caused by ARVs after 3 months’ follow-up. A 47.6% (129/271) had hepatitis C tests, and 2 patients were found with hepatitis C infection and abided by follow-up for 6 months.

For the follow-up assessment at week-4 to week-6 after exposure, 87.5% (237/271) received tests by the SD Bioline Syphilis/HIV Duo assay, and none of the patients seronegative at baseline had seroconverted for HIV and syphilis infections. A 55.4% (150/271) had tests for CBC, renal, and liver function, no abnormal outcomes and indicators were found.

As for the follow-up tests at 3-month and/or 6-month after exposure, 79.7% (216/271) had tests for both HIV and syphilis, 1 case of HIV seroconversion was reported. Documented information indicated this seroconversion patient was tested as negative at the 6-week after exposure though he discontinued medication 2 weeks later due to sex partner not living with HIV, however, he had ongoing high-risk behaviors (UAI, oral penetration) and was confirmed as positive at the 82nd day within the follow-up schedule, not ascribed as nPEP failure.

When it came to follow-up retention based on the definition of LTFU, our research realized an overall follow-up rate of 91.9% (249/271), with the calculation from 205 participants coming back to CBOs for HIV tests both at week-4 to week-6 and 3-month and/or 6-month after exposure, as well as 32 and 12 MSM, respectively had test only 1 time at week-4 to week-6, and at 3-month and/or 6-month after exposure. Likewise, the follow-up rate of participants completing a full 28-day medication reached 92.8% (231/249).

## 4 Discussion

HIV transmission persists, particularly in MSM who usually retain UAI behaviors, and some may experience condom failure even though kept condom use. As a result, nPEP is an imperative component of public health strategy for HIV prevention in this group. Using almost one and a half years of clinical data, we described nPEP practices in two cities of Southwestern China through partnerships with CBOs. Most participants prescribed with nPEP at ART clinics were young men (84.1%) potentially exposed to HIV through high-risk sexual behaviors, and a certain number of patients (32.8%) were identified as a sex role of receptive anal (“0”) which had been ranked the second for HIV transmission risk.^[45]^ Our research indicated 11.4% nPEP repeat presenters, similar to other studies,^[27,46]^ which highlight the importance of sexual health screenings and counseling on safe sex practices, especially in the MSM community, and additional attention should be paid to repeat presenters for risk compensation that risk behavior counseling by CBOs and considerations for pre-exposure prophylaxis (PrEP) as an alternative intervention tool by ART doctors should be encouraged, despite previously nPEP use may not correlate with increased high-risk behaviors among MSM population.^[39,47]^ PrEP has not been officially promoted across China but it is currently available at some ART clinics in pilot sites. Corresponding guideline suggests HIV-serodiscordant couples, nPEP repeat presenters as well as groups of high-risk behaviors including MSM, intravenous drug users, and individuals not in a mutually monogamous relationship.^[35]^

### 4.1 Medication uptake and SEs

A large majority of patients (93.3%) were prescribed nPEP within 48 hours. It is worth mentioning that 7.4% at 0:00 to 6:00 and 26.9% at 18:00 to 0:00 came to CBOs for nPEP counseling, which indicates the flexibility of working time by CBOs just meets the target population's requirement for services. Despite potential benefits, the provision of nPEP remains underutilized because of the cost to patients.^[48]^ In our study, 9 MSM gave up treatment since they could not afford the nPEP cost. Overall, half the participants selected the relatively inexpensive regimen of “TDF/FTC” ($ 286.96). Therefore, we suggest improve healthcare providers’ ability to navigate the systems required to have nPEP medications covered by insurance, which is particularly important to those most willing to use nPEP but with lower or no incomes, for example, student group. To the best of our knowledge, the regimen of E/C/F/TAF, of whom price decreased from $431.88 to $186.96 for a full course of nPEP since January 2020, had been covered by insurance, owing to drug negotiation between the National Healthcare Security Administration and manufacturers, quite inspiring news for domestic clients. A prospective trial found E/C/F/TAF was well-tolerated when used as an nPEP regimen.^[19]^

Our analysis revealed that 18 participants were with documented SEs and 3 of them discontinued treatments, mainly presented as mild gastrointestinal, neurologic disorders, and musculoskeletal pain but were well coped with, no serious adverse events occurred. It may remind us that such kind of SEs is common in ARVs, which can be tolerated by most nPEP users. A meta-analysis by Ford et al showed that SEs could lead to treatment interruption or non-compliance, thereby affecting the efficacy of nPEP.^[21]^ Their findings indicated a total of 1033 participants from 64% of the studies had terminated nPEP due to SEs. A systematic review in Nigeria also found 23.8% of the patients could not finish treatment induced by SEs.^[49]^ Treatment completion was associated with SEs in our study. All these suggest ongoing adherence education, medication guidance, and psychological supports are essential in the course of nPEP for avoiding non-compliance.

### 4.2 Adherence to nPEP and retention in follow-up

Our study achieved quite encouraging results on medication adherence, with an nPEP completion rate of 93.6%, higher than similar researches, as seen in findings from Belgium (60%–66.4%), America (64%), and Canada (49%).^[27,50–52]^ Our analysis by the multi-variate model highlighted that SEs were important considerations affecting nPEP compliance. In general, the HIV-testing follow-up of our research also demonstrated a higher rate (91.9%). The study aforementioned in Belgium found 41.1% failed to attend their follow-up schedule,^[50]^ the same as other similar studies, the follow-up rates just ranged from 30% to 60%.^[18,26,53,54]^ The efficacy of nPEP greatly relies on the compliance of patients to the regimen prescribed. In addition, ongoing follow-up consultations in the process of nPEP can bring about advantages beyond monitoring SEs and increasing adherence. Follow-up is of paramount importance as it is the key not only to offer an opportunity for confirming the results of nPEP protection but a chance for more counseling on subsequent risk behaviors. Furthermore, follow-up visits are considered as an occasion for discussing PrEP which might be the optimum therapy option for a subgroup of clients consulting for nPEP.

Our good outcomes in adherence and retention in care could be ascribed to nPEP publicities ahead of the recruitment as well as the “one-to-one” follow-up model by CBOs. Efforts contributed to publicizing nPEP knowledge by Nanning Yitongxing Healthcare Center and Liuzhou Hongying Working Group were particularly appreciated. An nPEP feuilleton through the WeChat public account (wwwnnchcn) of the Healthcare Center was established aiming at community publicity and advocacy; besides, nPEP information through QQ, Weibo, Blued (a strong social gay APP connecting gay communities around the globe) was conveyed, which helped MSM correctly grasp nPEP, urging them to complete the treatment. On the other hand, our lower drop-out rate would be inseparable from an LGBT-friendly environment amid the research. Despite the criminalization of homosexuality in China is not proactively enforced, discrimination against MSM hinders them to receive HIV-related service. However, MSM CBOs show their unique and irreplaceable strengths in regard to LGBT-based HIV prevention: Firstly, as a supplement to CDC and medical institutions, CBOs are regarded as a crucial bridge between HIV high-risk groups and authorities in health. Secondly, CBOs’ flexible working hours and diversified serving manners can satisfy the variable needs of target groups. Thirdly, most important CBOs share a common subculture with the LGBT community, which effectively alleviates stigma and discrimination against the homosexual population, thereby a trust patient–prescriber relationship being created and better reach out to the hidden groups.^[55,56]^ CBOs engagement in this MSM-oriented nPEP highlights such advantages. On the contrary, nPEP implemented independently by the Department of STI Control in Singapore indicated MSM has significantly associated with LTFU, hence correspondingly affected adherence.^[26]^ Taken together, potential models fitting the LGBT group ought to be explored just as a similar study suggests a “one-size-fits-all” model for nPEP is probably not effective for marginalized populations.^[28]^

### 4.3 HIV seroconversions

In this research, follow-up HIV assessments at week-4 to week-6 after exposure showed no seroconversions. One nPEP recipient was found subsequently seroconverted to HIV at 3-month after exposure due to ongoing high-risk behaviors, not ascribed as nPEP failure, as observed in other similar studies.^[51,57]^ There is something noticeable that 46 participants’ source contact had been confirmed as HIV positive, and 44 nPEP users were tested as negative during the repeating HIV serology, except for 2 not complying with the follow-up appointment. All again testify that nPEP, one of several biomedical HIV prevention strategies, is truly effective in real-world settings. From an epidemiological perspective, the provision of nPEP at the same time could help new HIV cases identification, and our results show that 5 HIV-infected MSM were discovered, including 4 at baseline and 1 during the service, which is in agreement with findings from a Cameroon review.^[58]^

### 4.4 Other laboratory testing

Since nPEP regimens may pose an inhibitory effect on the hepatitis B virus (HBV), recipients need to be tested for liver and kidney function, HBV, etc.^[34,59]^ However, our research findings demonstrated 82.3% had tests for CBC, renal, and liver function, and only 70.1%, 47.6%, respectively, received tests on HBV and hepatitis C virus. Data showed more disappointedly for the follow-up assessment at week-4 to week-6 after exposure, merely 55.4% had tests for CBC, renal, and liver function, in spite of obtaining $14.49 for transportation subsidy with the presentation of test reports. To our understanding, this part had not been underscored before nPEP as well as counseling in the service. Although there was no liver and kidney damage caused by ARVs reported in our research, it does not mean there is no risk, which reminds the Chinese nPEP guideline makers should make much account of such tests in the future.

### 4.5 Limitations

There were several crucial limitations in this study. On the one hand, our research was confined to participants’ self-report to assess nPEP use, instead of using validated clinical records, including data on source contact and HIV status, medication adherence, and side effects self-reported by patients in the process of consultation, subjected to self-reporting bias. On the other hand, our study showed that side effect was an essential element associated with adherence to nPEP. However, we could not rule out the existence of unmeasured confounders, for instance, education level and income failed to be captured as socio-demographic factors. Additionally, the study was well laid out at the beginning with strong support by both domestic and international cooperative projects. Hence, real conditions should be deliberated when the study findings are extrapolated to other settings in China.

## 5 Conclusions

Through partnerships with CBOs, our pilot study on nPEP aiming at MSM group from September 2017 to December 2019 in two cities of Southwestern China revealed three important findings. First of all, CBOs’ engagement in HIV nPEP, especially the “one-to-one” follow-up supports by peer educators partly ensure medication compliance and follow-up retention. This collaborative model would be encouraged to other places where conditions permitted, in particular China where the “China AIDS Fund for Non-Governmental Organizations” has been officially launched since 2015. Second, adequate publicities on nPEP knowledge could be conducive to the efficacy of post-exposure prophylaxis. The role of CBOs should be highlighted in publicity and advocacy. Third, from the perspective of providers, tailored interventions are needed to address the subsequent high-risk behaviors among MSM.

## Acknowledgments

The authors would like to thank all staff members from U.S. CDC Global AIDS Program, China Office, Guangxi CDC, Nanning and Liuzhou CDCs, Nanning the Fourth People's Hospital, Liuzhou People's Hospital, and Guangxi Longtan Hospital, in particular the peer educators from Nanning Yitongxing Healthcare Center and Liuzhou Hongying Working Group for involving in the research. We thank all our participants for their time. Besides, we would like to deliver thanks to QGH from Guangxi Medical University for assistance with the statistical analysis.

## Author contributions

GHL, YFW, QYZ, SJL, and RJL conceived and designed the study. YFW conducted data processing and statistical analysis as well as wrote the manuscript. GHL revised the manuscript. We further confirm that the order of authors listed in the manuscript has been approved by all of us. All authors read and approved the final manuscript.

**Conceptualization:** Yufei Wu, Qiuying Zhu, Shujia Liang, Rongjian Li, Guanghua Lan.

**Data curation:** Yufei Wu, Yuejiao Zhou.

**Formal analysis:** Yufei Wu.

**Funding acquisition:** Guanghua Lan.

**Investigation:** Guanghua Lan, Yufei Wu, Qiuying Zhu, Nengxiu Liang.

**Methodology:** Yufei Wu.

**Project administration:** Guanghua Lan.

**Software:** Chunying Li.

**Supervision:** Yufei Wu, Guanghua Lan.

**Validation:** Guanghua Lan.

**Writing – original draft:** Yufei Wu.

**Writing – review & editing:** Guanghua Lan, Yufei Wu.
